# Exploring the potential mechanisms of Erigeron breviscapus in the treatment of glaucoma based on network pharmacology and molecular docking

**DOI:** 10.1097/MD.0000000000043970

**Published:** 2025-08-15

**Authors:** Enyang Yang, Yuehong Zhu, Xuelei Chen, Xinyi Xie, Zhihong Ma

**Affiliations:** a Huzhou Nanxun People’s Hospital, Huzhou, China; b Huzhou Key Laboratory of Molecular Medicine, Huzhou Central Hospital, Fifth School of Clinical Medicine of Zhejiang Chinese Medical University, Huzhou, China.

**Keywords:** AKT1, *Erigeron breviscapus*, glaucoma, molecular docking, network pharmacology, therapy target

## Abstract

To elucidate the mechanism of action of *Erigeron breviscapus* (*EB*) in the treatment of glaucoma, we employed a comprehensive approach combining network pharmacology and molecular docking. The primary active compounds and corresponding targets of *EB* were identified utilizing the Traditional Chinese Medicine Systems Pharmacology (TCMSP) database. Concurrently, glaucoma-related disease targets were sourced from the GeneCards database. The intersection of drug and disease targets was determined using Venny 2.1.0. Potential targets of *EB* for glaucoma treatment were further explored by constructing protein–protein interaction networks via the STRING database and analyzed using Cytoscape 3.8.0 software. Finally, the validation of protein molecular docking was conducted using AutoDockTools and the PyMOL software. The *EB* screening identified 12 active ingredients and 161 gene targets for glaucoma treatment. Protein–protein interaction network analysis highlighted core targets like AKT serine/threonine kinase 1, estrogen receptor 1, epidermal growth factor receptor, and others, involved in cell signaling, neovascularization, cell proliferation, apoptosis inhibition, and inflammation. Gene ontology and Kyoto Encyclopedia of Genes and Genomes pathway analyses revealed that *EB* treatment for glaucoma primarily affects signaling pathways related to oxygenated compounds, cell proliferation, cell communication, protein kinase and oxidoreductase activities, PI3K-AKT, RAS, and VEGF. Further molecular docking indicated that EB’s active ingredients effectively bind to the key target AKT1. In conclusion, the network pharmacology approach identified the drug-active-ingredient-target-disease interactions of *EB* for glaucoma, predicting gene targets and signaling pathways. This offers a foundation for understanding *EB*’s mechanism and aids in glaucoma drug development.

## Graphical abstract

**Figure:**
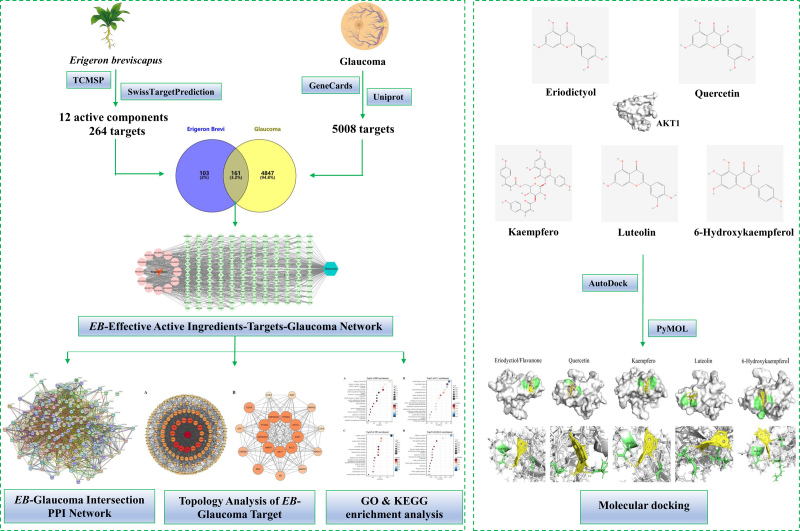

## 1. Introduction

Glaucoma is an irreversible blinding ophthalmic disease caused by a variety of factors. The main cause of glaucoma is the progressive impairment of visual function and optic nerve atrophy due to pathologically high intraocular pressure, which leads to the narrowing of the patient’s field of vision and even blindness.^[1,2]^ Currently, the primary therapeutic approach for glaucoma involves the reduction of intraocular pressure and the preservation and restoration of optic nerve function.^[3,4]^ In clinical practice, western medical and surgical interventions have demonstrated effectiveness in lowering intraocular pressure in patients with glaucoma.^[5,6]^ However, western medicine and surgical treatments are more destructive and riskier, and their overall efficacy needs improvement. In recent years, with the development of Traditional Chinese Medicine (TCM) theory and practice, the advantages of TCM in the treatment of glaucoma have been widely recognized.^[7]^

*Erigeron breviscapus* (*EB*), also known as Dandelion Flower, is the dried whole herb of the Asteraceae plant. Previous studies have shown that *EB* has the effects of dilating blood vessels, improving microcirculation, increasing blood flow, expanding the visual field, and protecting retinal ganglion cell damage in rats caused by elevated intraocular pressure.^[8–10]^ However, little is known about the efficacy and mechanism of action of *EB* in treating glaucoma lesions. This study employs network pharmacology to screen and identify the targets of *EB* in the treatment of glaucoma, providing a theoretical basis for the clinical application and fundamental research of *EB* in treating glaucoma.

## 2. Materials and methods

This study does not need ethical approval because there is no personal data involved.

### 2.1. Active compounds and corresponding targets in EB

The principal active compounds of *EB* were identified using the Traditional Chinese Medicine Systems Pharmacology Database and Analysis Platform (TCMSP; https://www.tcmsp-e.com/?q=targets#/database). The selection criteria for effective active compounds included an oral bioavailability of ≥30% and a drug-likeness score of ≥0.18. Subsequently, the SwissTargetPrediction platform (http://swisstargetprediction.ch/index.php) was employed to determine the drug targets associated with these effective active ingredients.

### 2.2. Glaucoma-related targets

Access the GeneCards genome annotation database (https://www.genecards.org/), input the keyword “glaucoma” into the human gene database, and export the associated disease targets related to glaucoma in Excel format.

### 2.3. The intersection target of EB and glaucoma

Venny2.1.0 was employed to identify the intersection between the targets of the active components of *EB* and the disease targets associated with glaucoma, thereby identifying the potential targets of *EB*’s effective active components in the treatment of glaucoma.

### 2.4. Protein–protein interaction (PPI) networks construction

The network was constructed using the STRING online tool (https://cn.string-db.org). PPI with a minimum required interaction score >0.4 were selected, and isolated nodes were excluded. The PPI graph was downloaded and saved in.tsv format. The.tsv file obtained from STRING was subsequently processed using Cytoscape 3.8.0 software. The “cytoNCA” plugin was employed to calculate the network parameters, and the values of Degree, betweenness centrality (BC), and closeness centrality were utilized as criteria for identifying the core targets.

### 2.5. Gene ontology (GO) enrichment analysis and Kyoto Encyclopedia of Genes and Genomes (KEGG) pathway enrichment analysis

The DAVID database (https://david.ncifcrf.gov/tools.jsp) was utilized to conduct Gene Ontology (GO) enrichment analysis and Kyoto Encyclopedia of Genes and Genomes (KEGG) pathway enrichment analysis on the targets associated with the active components of *EB*, as well as the common targets related to glaucoma. A significance threshold of *P* < .05 and a false discovery rate <.05 was applied to predict the potential targets of *EB* in the treatment of glaucoma. The results were presented in the form of bubble plots.

### 2.6. EB -active ingredient-target- pathway network construction

Through KEGG pathway enrichment analysis, the main pathways and corresponding target genes of *EB*’s effect on glaucoma were obtained. These were imported into Cytoscape 3.8.0 for visualization, resulting in a network diagram of herbs-compounds-targets-pathways. The network diagram was then analyzed to identify the pathways with significant effects, which were considered the main signaling pathways for *EB*’s treatment of glaucoma.

### 2.7. Molecular docking

The active compounds identified in the TCMSP database were retrieved from the PubChem database (https://pubchem.ncbi.nlm.nih.gov/). The target protein structure was obtained from the PDB database (https://www.rcsb.org/) and subsequently imported into PyMOL and AutoDock Tools software for format conversion, removal of water molecules, separation of ligands, addition of hydrogen atoms, and charge assignment. Molecular docking was conducted using AutoDock software to visualize the docking interactions.

## 3. Results

### 3.1. Screening of effective active components and targets of EB

Utilizing the TCMSP analysis platform, a total of 50 types of active components of *EB* were identified. Based on the screening criteria of oral bioavailability ≥30% and drug-likeness ≥0.18, 12 effective active components of *EB* were selected, as detailed in Table 1. Following data validation and refinement through the Uniprot database, 264 targets were identified. The 6 active ingredients with the highest number of targets were luteolin, quercetin, kaempferol, naringenin, 6-hydroxykaempferol, and baicalein.

**Table 1:** The effective active ingredients in *EB* and their pharmacokinetic parameters.

Mol ID	Effective active ingredients	Oral bioavailability (%)	Pharmacological properties
MOL007963	1-Hydroxy-2,3,5-trimethoxy xanthone	101.06	0.30
MOL000392	Formononetin	69.67	0.21
MOL002712	6-Hydroxykaempferol	62.13	0.27
MOL000098	Quercetin	46.43	0.28
MOL000816	Ergosta-7,22-dien-3-one	44.88	0.72
MOL007984	Δ5,22-Stigmastadien-3-ol	43.83	0.76
MOL005922	Acanthoside B	43.35	0.77
MOL001040	((2*R*)-5,7-Dihydroxy-2-(4-hydroxyphenyl)chroman-4-one)	42.36	0.21
MOL000422	Kaempferol	41.88	0.24
MOL002914	Eriodyctiol/flavanone	41.35	0.24
MOL000006	Luteolin	36.16	0.25
MOL002714	Baicalein	33.52	0.21

### 3.2. Acquisition of glaucoma-related targets

The GeneCards database was utilized to identify 6848 gene targets associated with glaucoma. Following validation and correction using the Uniprot database, a total of 5008 human glaucoma-related targets were confirmed.

### 3.3. EB- glaucoma common targets

**Figure 1.**
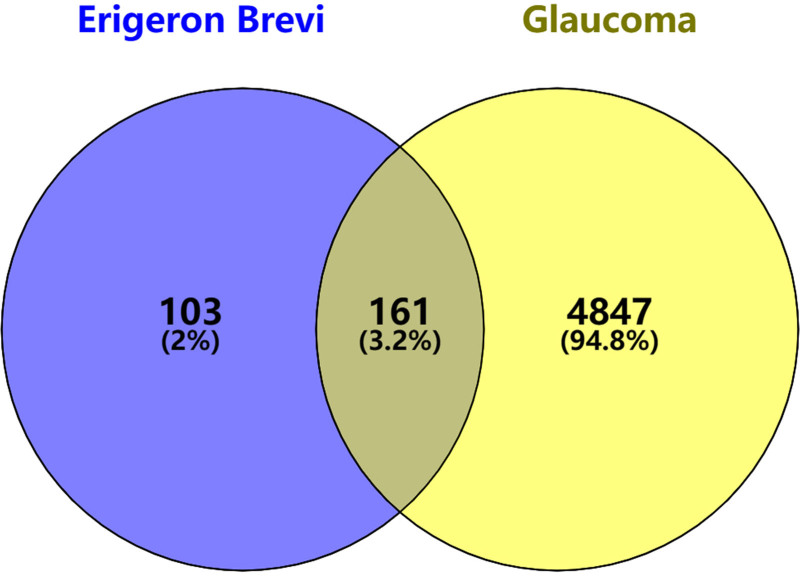
Target screening of *EB* and glaucoma. *EB* = Erigeron breviscapus.

An intersection of 264 therapeutic targets associated with *EB* components and 5008 disease targets related to glaucoma were identified, resulting in the identification of 161 potential *EB* targets for the treatment of glaucoma, as illustrated in Figure 1.

### 3.4. Construction of “drug-active ingredient-target-disease” network

**Figure 2.**
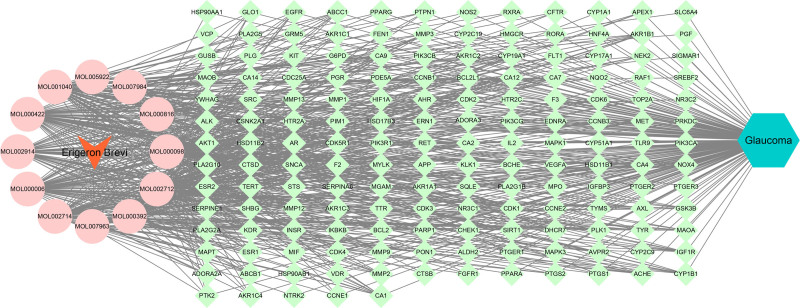
*EB*-Effective active ingredients-targets-glaucoma network. The reddish-orange arrows represent *EB*, the fractional circles represent active ingredients, the grass-green rectangles represent common targets, and the cyan-blue hexagons represent diseases. *EB* = Erigeron breviscapus.

The active ingredients of *EB* and the intersecting targets associated with glaucoma were compiled into an Excel spreadsheet and subsequently imported into Cytoscape 3.8.0 software. This process facilitated the construction of a network diagram illustrating the relationships among the drug-active ingredients-targets-disease, as depicted in Figure 2.

### 3.5. Construction of drug-disease target PPI network

**Figure 3.**
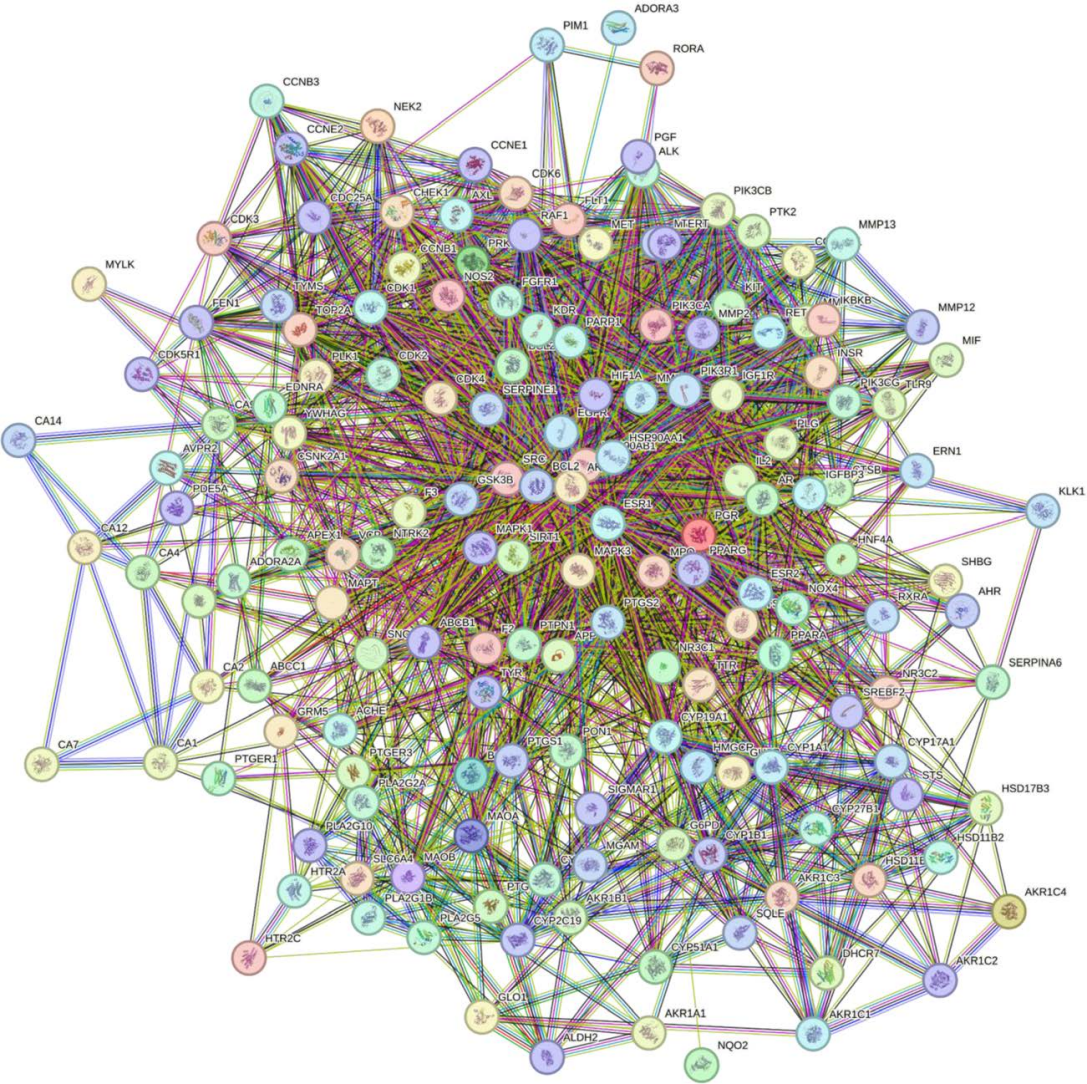
*EB*-glaucoma intersection PPI network. *EB* = Erigeron breviscapus, PPI = protein–protein interaction.

**Figure 4.**
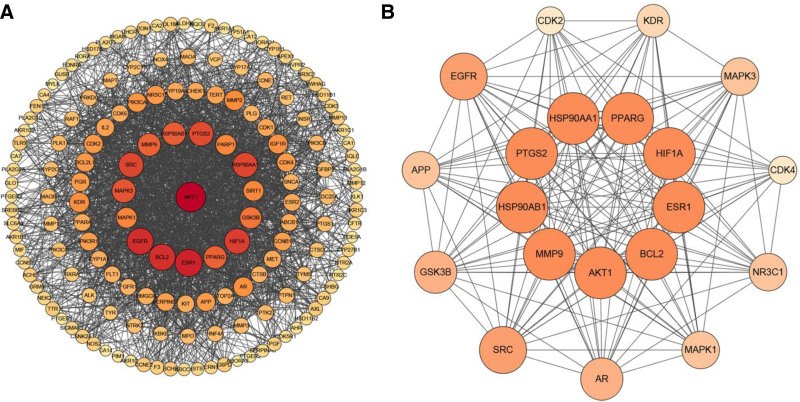
*EB*-glaucoma target and hub gene topology analysis. (A) 161 intersecting targets for *EB* treatment of glaucoma from Cytoscape. (B) The top 20 hub genes for *EB* treatment of glaucoma from Cytoscape. *EB* = Erigeron breviscapus.

The 161 targets were input into the STRING online tool, with the interaction threshold confidence set to a minimum of 0.4, to construct the protein–protein interaction (PPI) network diagram. This process resulted in the identification of 161 nodes and 799 edges, yielding an average node degree of 25.6 (see Fig. 3). In this network, nodes represent the predicted targets, while edges denote the interactions between proteins. The PPI network diagram generated by the STRING platform was subsequently downloaded and imported into Cytoscape version 3.8.0 for enhanced visualization. The Analyze Network plug-in was employed to determine the degree value of the network nodes, resulting in the construction of the PPI topology diagram (Fig. 4A) through degree adjustment. In this diagram, larger nodes with deeper colors signify more critical targets. As shown in Figure 4A, the top ten targets for *EB* treatment of glaucoma include AKT serine/threonine kinase 1 (AKT1), estrogen receptor 1 (ESR1), epidermal growth factor receptor (EGFR), BCL2 apoptosis regulator (BCL2), hypoxia inducible factor 1 subunit alpha (HIF1A), SRC proto-oncogene, non-receptor tyrosine kinase (SRC), heat shock protein 90 alpha family class a member 1 (HSP90AA1), prostaglandin-endoperoxide synthase 2 (PTGS2), mitogen-activated protein kinase 3 (MAPK3), and heat shock protein 90 alpha family class b member 1 (HSP90AB1). Subsequently, the CytoHubba plugin was used to identify common targets among the top 30 targets of BC, closeness centrality, and Degree Centrality, and these targets were designated as core targets. This analysis identified a total of 20 core targets: AKT1, ESR1, EGFR, BCL2, HIF1A, HSP90AA1, SRC, PTGS2, MAPK3, HSP90AB1, peroxisome proliferator activated receptor gamma, matrix metallopeptidase 9 (MMP9), glycogen synthase kinase 3 beta (GSK3B), mitogen-activated protein kinase 1, kinase insert domain receptor (KDR), androgen receptor, Nuclear receptor subfamily 3 group c member 1 (NR3C1), cyclin-dependent kinase 2, amyloid beta precursor protein (APP), and cyclin-dependent kinase 4. After analyzing these core targets on the STRING platform, the data was imported into Cytoscape 3.8.0 software for further inspection, generating topology map (Fig. 4B).

### 3.6. GO functional enrichment analysis and KEGG target pathway enrichment analysis

The drug-disease intersection targets were analyzed using Sangerbox (http://sangerbox.com/) for GO and KEGG pathway enrichment. This yielded 3508 significant GO entries (*P* < .05), comprising 2967 biological processes (BP), 205 cellular component (CC), and 336 molecular functions (MF). These entries were ranked by *P* value, with lower values indicating higher enrichment.

**Figure 5.**
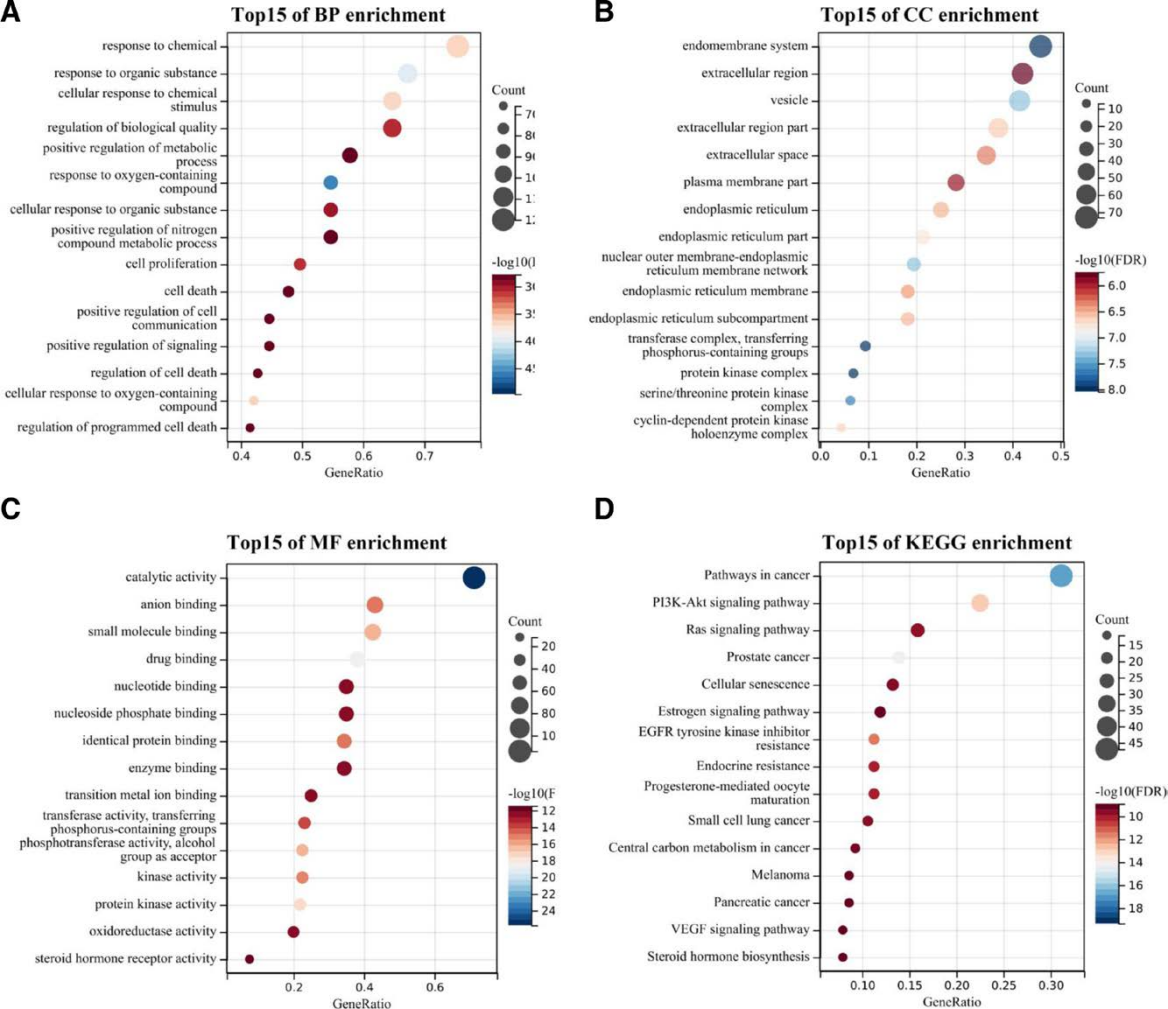
GO enrichment analysis and KEGG enrichment analysis of *EB* treatment for glaucoma. (A) The top 15 significant biological process (BP), (B) the top 15 significant cellular component (CC), and (C) the top 15 significant molecular function (MF) of GO enrichment; (D) KEGG pathway enrichment analysis of the cross targets. BP = biological processes, CC = cellular component, *EB* = Erigeron breviscapus, GO = gene ontology, KEGG = Kyoto Encyclopedia of Genes and Genomes, MF = molecular functions.

Items were sorted by *P* value, which was inversely related to enrichment level. A bubble diagram displayed the top 15 enriched items in BP, CC, and MF. GO functional enrichment analysis revealed that BP was primarily associated with responses to oxygen-containing compound, responses to organic substance, cellular response to chemical stimulus, response to chemical, cellular response to oxygen-containing compound, etc (Fig. 5A); CC mainly focused on transferase complex, transferring phosphorus-containing groups, protein kinase complex, endomembrane system, serine/threonine protein kinase complex, vesicle, nuclear outer membrane-endoplasmic reticulum membrane network, extracellular region part, and endoplasmic reticulum membrane, etc (Fig. 5B); MF mainly focuses on catalytic activity, drug binding, protein kinase activity, small molecule binding, phosphotransferase activity, and alcohol group as acceptor, etc (Fig. 5C).

KEGG enrichment analysis obtained 148 pathways that are important for *EB* treatment of glaucoma. The main pathways are closely related to the pathways in cancer, prostate cancer, PI3K-Akt signaling pathway, EGFR tyrosine kinase inhibitor resistance, endocrine resistance, etc (Fig. 5D).

### 3.7. Molecular docking

**Figure 6.**
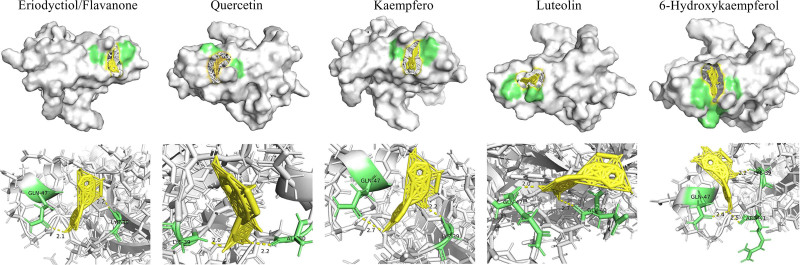
Molecular docking model of AKT1 with flavanone, quercetin, kaempferol, luteolin, and 6-hydroxykaempferol.

Molecular docking stability is indicated by binding energy; lower values suggest greater stability and less energy needed for binding. Binding energy below −1.2 kcal/mol signifies good activity. The top 1 core target gene AKT1 and its potential active compounds underwent docking, resulting in binding energies of −5.3 kcal/mol for flavanone, −4.06 kcal/mol for quercetin, −4.94 kcal/mol for kaempferol, −4.04 kcal/mol for luteolin, and −4.78 kcal/mol for 6-hydroxykaempferol, respectively (Fig. 6).

## 4. Discussion

Network pharmacology is a branch of science that, based on the theoretical foundations of systems biology and multidirectional pharmacology, utilizes biomolecular network analysis methods to examine the effective components and their synergistic effects in TCM prescriptions or single compounds from both molecular and systemic perspectives.^[11]^ It scientifically elucidates the pharmacological mechanisms of TCM. Built on the interaction network of “disease-gene-target-drug,” it emphasizes analyzing the molecular correlation patterns between drugs and their therapeutic targets from a systemic and holistic.^[11–14]^

Modern pharmacological studies indicate that *EB* has pharmacological effects such as vasodilation, improvement of microcirculation, and enhancement of blood supply to the heart and brain; regulation of blood lipids, reduction of blood viscosity, and improvement of blood rheology; inhibition of platelet and red blood cell aggregation, promotion of fibrinolytic activity; scavenging of oxygen free radicals, combating lipid peroxidation, and ischemia-reperfusion injury.^[4,9,15]^ With the rapid development and innovation of modern information technology and chromatographic techniques, the main chemical components of *EB* have been gradually revealed and elucidated. Caffeoyl esters and flavonoids are the main chemical components of *EB*.^[16–18]^ This study identified 50 active components through TCMSP screening, including quinic acid, ethyl caffeate, and methyl caffeate, all of which are caffeoyl ester compounds. Among the 12 effective active components, most are flavonoids, such as luteolin, quercetin, kaempferol, naringenin, 6-hydroxykaempferol, and baicalein. Based on network pharmacology, we identified the action targets of 12 active components and the disease targets of glaucoma, and then found the intersection, resulting in a total of 161 targets for *EB* in the treatment of glaucoma. By constructing the drug-active ingredient-target-disease network diagram, it can be seen that *EB* exhibits a multi-component, multi-target action mode in the treatment of glaucoma. By establishing a PPI network through String, it was found that the core targets of *EB* in the treatment of glaucoma include several cell signaling pathway proteins such as phosphorylated protein kinase AKT1, estrogen receptor (ESR1), HSP90AB1, neuroprotective factor amyloid precursor APP, HIF1A, peroxisome proliferator activated receptor gamma, SRC, mitogen-activated protein kinase 1, MAPK3, and some proteins that promote angiogenesis, promote cell proliferation, and inhibit apoptosis, such as EGFR,VEGFR-2, KDR, cyclin-dependent kinase 2, cyclin-dependent kinase 4, transcription factor NR3C1, BCL2, HSP90AA1, GSK3B, and some inflammatory factors such as PTGS2, MMP9, etc. These results suggest that *EB* may protect or restore optic nerve function in the treatment of glaucoma by promoting cell proliferation, inhibiting apoptosis.

To investigate how *EB* treats glaucoma, GO and KEGG analyses were conducted. GO analysis highlighted functions like reactions with oxygen-containing compounds, cell proliferation, programmed cell death regulation, and various enzyme activities. KEGG analysis indicated involvement of the PI3K-AKT pathway, resistance to EGFR tyrosine kinase inhibitors, Ras signaling, and VEGF signaling. pathway. Relevant studies have also shown that PI3K-AKT signaling pathway and VEGF signaling pathway can participate in the occurrence and development of glaucoma.^[19–22]^

In this study, AKT1, predicted to be the most critical target gene for *EB* treatment of glaucoma, was subjected to molecular docking validation with its binding chemical components. The binding energies of AKT1 with flavonoids, quercetin, kaempferol, luteolin, and 6-hydroxykaempferol are all below −1.2 kcal/mol, indicating a good binding effect (Fig. 6). AKT1, a serine/threonine protein kinase from the AKT family, is linked to the PI3K-AKT pathway, influencing cell proliferation, apoptosis, and glucose metabolism.^[22,23]^ This study identified AKT1 as a target of *EB*’s active ingredients, with GO and KEGG analyses confirming its crucial role in glaucoma treatment. CytoHubba and molecular docking results further suggest AKT1’s significant involvement in *EB*’s therapeutic effects on glaucoma.

## 5. Conclusions

In summary, this study, based on network pharmacology theory, analyzed the effective active components of *EB* and the potential action targets for treating glaucoma. Through GO and KEGG enrichment analysis, it further explored the complex functions and possible pathways of *EB* in treating glaucoma, indicating that *EB* treatment of glaucoma is a complex process involving multiple components, multiple target actions, and multi-pathway coordination. However, due to the limitations in our understanding of glaucoma and in network pharmacology research, the aforementioned results still require further validation through related experiments.

## Acknowledgments

We are very grateful for the contributions of the TCMSP, SwissTargetPrediction, GeneCards, STRING, DAVID, PubChem, PDB, Sangerbox databases, which provide information on this research, as well as all colleagues involved in the study.

## Author contributions

**Data curation:** Zhu Yuehong.

**Project administration:** Zhihong Ma.

**Software:** Xinyi Xie.

**Validation:** Xuelei Chen.

**Writing – original draft:** Yang Enyang.

**Writing – review & editing:** Zhihong Ma.
